# Determinants of whole-body maximal aerobic performance in young male and female athletes: The roles of lower extremity muscle size, strength and power

**DOI:** 10.1371/journal.pone.0262507

**Published:** 2022-01-12

**Authors:** Takaki Yamagishi, Akira Saito, Yasuo Kawakami

**Affiliations:** 1 Faculty of Sport Sciences, Waseda University, Saitama, Japan; 2 Department of Sport Science, Japan Institute of Sport Sciences, Tokyo, Japan; 3 Center for Health and Sports Science, Kyushu Sangyo University, Fukuoka, Japan; 4 Human Performance Laboratory, Comprehensive Research Organization, Waseda University, Tokyo, Japan; Universita degli Studi di Milano, ITALY

## Abstract

This study sought to determine whether lower extremity muscle size, power and strength could be a determinant of whole-body maximal aerobic performance in athletes. 20 male and 19 female young athletes (18 ± 4 years) from various sporting disciplines participated in this study. All athletes performed a continuous ramp-incremental cycling to exhaustion for the determination of peak oxygen uptake ($\dot{V}O_{2peak}$: the highest $\dot{V}O_{2}$ over a 15-s period) and maximal power output (MPO: power output corresponding to $\dot{V}O_{2peak}$). Axial scanning of the right leg was performed with magnetic resonance imaging, and anatomical cross-sectional areas (CSAs) of quadriceps femoris (QF) and hamstring muscles at 50% of thigh length were measured. Moreover, bilateral leg extension power and unilateral isometric knee extension and flexion torque were determined. All variables were normalised to body mass, and six independent variables ($\dot{V}O_{2peak}$, CSAs of thigh muscles, leg extension power and knee extension and flexion torque) were entered into a forward stepwise multiple regression model with MPO being dependent variable for males and females separately. In the males, $\dot{V}O_{2peak}$ was chosen as the single predictor of MPO explaining 78% of the variance. In the females, MPO was attributed to, in the order of importance, $\dot{V}O_{2peak}$ (p < 0.001) and the CSA of QF (p = 0.011) accounting for 84% of the variance. This study suggests that while oxygen transport capacity is the main determinant of MPO regardless of sex, thigh muscle size also has a role in whole-body maximal aerobic performance in female athletes.

## Introduction

Maximal or peak oxygen uptake ($\dot{V}O_{2\max}/\dot{V}O_{2peak}$) is defined as the highest rate at which oxygen can be taken up and utilised by the body during whole-body maximal exercise [1]. Since $\dot{V}O_{2\max}$ is largely limited by the capacity of the cardiorespiratory system to transport oxygen to the exercising muscles [1–3], it is commonly employed to assess the cardiorespiratory capacity of individuals with diverse backgrounds [1, 4]. It has been suggested that the cardiorespiratory capacity sets the upper limit of endurance performance [1]. Indeed, Jacobs et al. [5] demonstrated that maximal aerobic cycling performance (maximal incremental power output) in highly trained endurance athletes was primarily attributed to the parameters of oxygen transport ($\dot{V}O_{2\max}$ and total body haemoglobin mass). Likewise, $\dot{V}O_{2\max}$ was chosen as one of the main predictors of maximal aerobic running performance (maximal incremental running velocity) in recreational runners [6]. On the other hand, muscle oxidative phosphorylation capacity has been shown to exceeds the upper limit of cardiorespiratory capacity during whole-body maximal exercise [7]. Moreover, elite endurance athletes do not necessarily show greater maximal arteriovenous oxygen difference compared with less-trained individuals [8]. Instead, they generally show an augmented cardiac output than less-trained counterparts during graded exercise [8, 9] due to an enhanced ability of increasing or maintaining stroke volume (SV) against increasing work rate [10]. While the peripheral factors such as muscle oxidative capacity should not be downplayed as they would particularly play an important role in submaximal endurance performance [1, 11, 12], the findings to date indicate that the capacity of cardiorespiratory system rather than that of oxygen extraction and/or utilisation seems to be a decisive factor in maximal aerobic performance.

Among various exercise modalities, incremental cycling is frequently utilised for the determination of maximal aerobic performance [5, 13, 14] due to its safe and easily standardised characteristics. However, compared with other exercise modalities (e.g. running), it could be assumed that greater load is imposed on specific muscle groups (e.g. thigh muscles) during the incremental cycling [15]. Indeed, energy produced by the knee extensors has been shown to account for the largest proportion (39%) of the work completed during cycling, followed by hip extensors (27%), ankle planter flexors (20%), knee flexors (10%) and hip flexors (4%) [16]. Furthermore, it has been suggested that quadriceps muscle group is largely responsible for the downstroke phase which corresponds to the phase of power generation [17]. In line with this suggestion, cross-sectional area (CSA) of knee extensors as well as maximal force in isometric half squat significantly correlated with Wingate peak power output in well-trained cyclists (r = 0.69 and 0.70, respectively) [18]. Moreover, it has been reported that the curvature constant (W′; a constant amount of work that can be performed above critical power) of the power-duration relationship has an impact on maximal power output during incremental cycling [19]. In addition, W′ has been associated with thigh CSA [20], thigh volume [21] and maximal torque of knee extensors [21]. Therefore, it can be argued that the parameters associated with power production of quadriceps muscle group or lower extremity not only explain short-term maximal cycling performance but also determine the limit of tolerance during exhausting cycling [17]. Nevertheless, a contribution from lower-extremity muscle size, strength or power to maximal aerobic cycling performance has never been directly verified.

Consequently, this study aimed to comprehensively determine the influence of lower extremity muscle size, strength and power on maximal aerobic cycling performance in male and female athletes. To accurately assess ones’ aerobic performance, these factors should be taken into consideration. It was hypothesised that such influence could become important for less endurance-trained athletes as they would experience more difficulty in supplying oxygen to the working muscles at higher intensities during the incremental cycling [8, 9, 22].

## Materials and methods

### Study design

Each athlete reported to the laboratory having only consumed water 4 h prior to arriving at the lab. Anthropometric and physiological measurements were performed in the following order: body composition, muscle morphology via magnetic resonance imaging (MRI), unilateral isometric knee extension and flexion torque, bilateral leg extension power and ramp incremental cycling to exhaustion. They were asked to refrain from any form of intense physical activity for 24 hours prior to a visit to the laboratory.

### Participants

Thirty-nine Japanese athletes (males: n = 20, 19 ± 3 years; females: n = 19, 18 ± 5 years, no age difference between the sexes, p = 0.584) participated in this study. Female athletes were asked to complete a self-reported questionnaire regarding their menstrual cycle phases (i.e. the first day of the last menstrual period prior to the measurement). We could obtain the completed questionnaires from 14 out of 19 athletes, and the phases of their cycles were as follows: 1 early follicular, 3 late follicular, 1 ovulatory, 3 early luteal, and 4 late luteal phases [23]. In addition, two of the female athletes answered that they had irregular menstrual cycles, whereas none of them reported the usage of oral contraceptive pills. Although we relied on the retrospective questionnaire and did not directly measure the fluctuations of the related sex hormones, Mattu et al. [24] recently showed that menstrual and contraceptive cycle phases did not affect submaximal ($\dot{V}O_{2}$ kinetics, maximal lactate steady state) and maximal ($\dot{V}O_{2\max}$ and time to exhaustion) parameters during cycling in healthy active women. Therefore, it was assumed that the differences in menstrual cycle phases among the female athletes would not have a major impact on the overall results of the current study. All athletes were selected by a local governing body during the study period, and they represented their county (Saitama prefecture) to compete in national and international sporting events of their age categories. Most athletes were competing at international level at the time of the study, and all were selected by the local governing body with the expectation that they would compete in the 2020 Tokyo Summer Olympics (five out of the 39 athletes competed in the 2020 Tokyo Olympics and two of them won a medal). The measurements of the current study were performed between July 2016 to February 2017. Sporting disciplines varied among athletes (Table 1), while years of experience in the selected sporting disciplines were significantly longer in male than female athletes (12 ± 3 vs. 9 ± 3 years, p = 0.005). All participants were fully informed both verbally and in writing about the study before giving their written informed consent. Informed consent from a guardian was also provided if a participant was under 20 years old. The study was approved by the Institutional Human Research Ethics Committee (IRB approval number: 2016–053) and carried out in line with the Declaration of Helsinki in 2013.

**Table 1:** Sporting disciplines of athletes.

Sports	Male athletes (n = 20)	Female athletes (n = 19)
Archery	0	1
Artistic swimming	0	2
Badminton	4	4
Basketball	0	3
Boxing	0	1
Cycling	0	1
Handball	1	0
Hockey	2	0
Ice hockey	1	0
Sport climbing	2	2
Swimming	6	3
Triathlon	1	0
Volleyball	0	1
Water polo	2	0
Wrestling	1	1

### Procedures

#### Body composition

The body mass, fat percentage and stature of each athlete were recorded to the nearest 0.1kg, 0.1% and 0.1cm, respectively. Body mass and fat percentage were measured on a bioelectrical impedance meter (HBF-701, Omron Corporation, Kyoto, Japan), while stature was measured using a stadiometer (YG-200, Yagami INC, Nagoya, Japan).

#### Measurement of cross-sectional areas of thigh muscles

**Fig 1:**
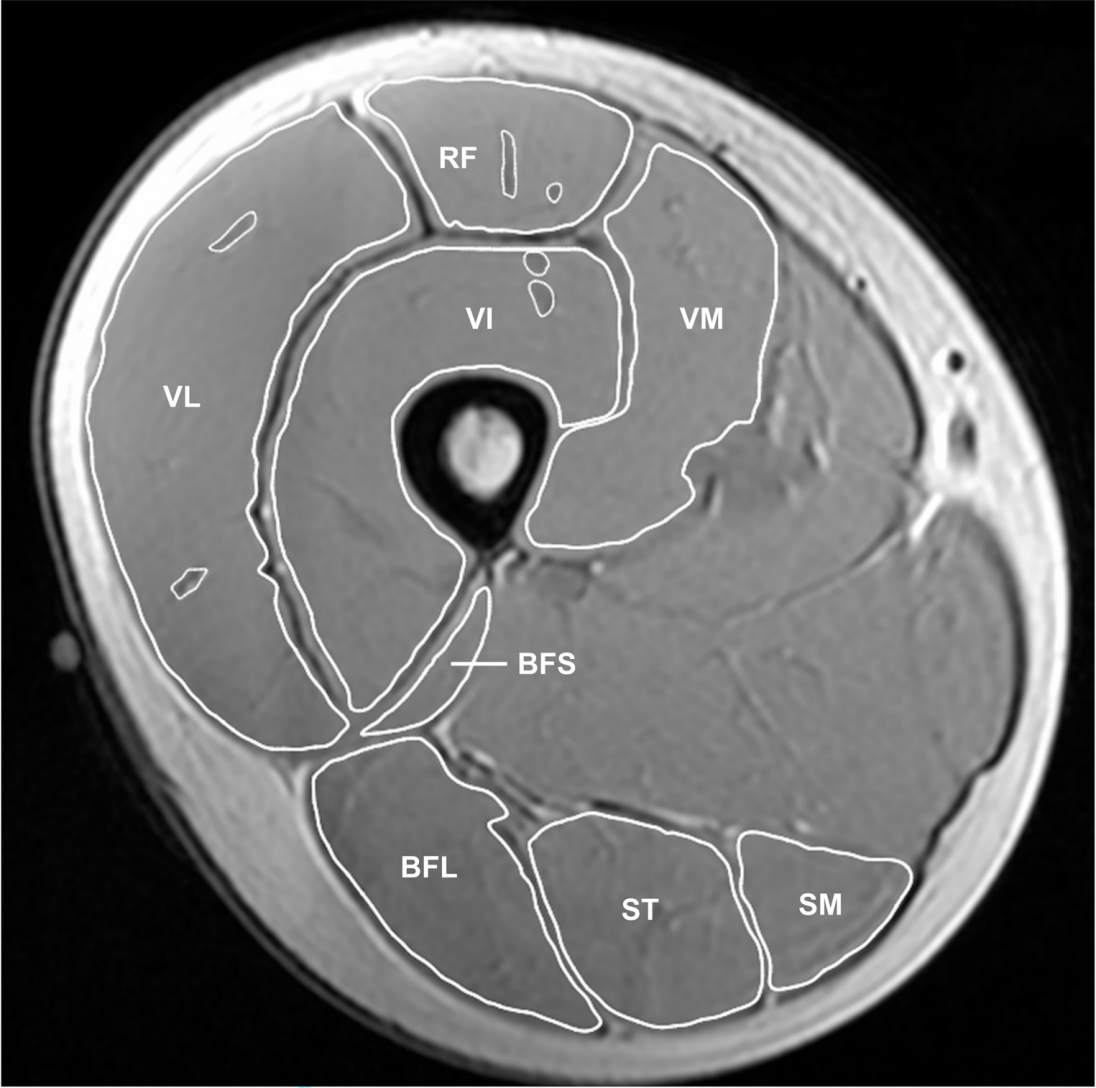
A typical example of 3D T1-weighted MR image at 50% of thigh length. RF = rectus femoris, VL = vastus lateralis, VM = vastus medialis, VI = vastus intermedius, BFS = biceps femoris short head, BFL = biceps femoris long head, ST = semitendinosus, SM = semimembranosus.

Series of cross-sectional images of the right leg were acquired using an MR scanner with an 8-ch body coil (Signa EX-CITE 1.5T, GE Medical systems, Chicago, USA). 3D T1-weighted fast spin gradient echo with the following parameters was utilised; echo time: 5.3 ms, repetition time: 11.8 ms, slice thickness: 8 mm, gap: 4 mm, matrix: 256 x 256, field of view: 260 mm x 260 mm. Participants lay supine with their arms and legs fully extended and relaxed in the magnet bore. Scanned MR images were transferred to a computer to measure anatomical cross-sectional areas (CSAs) of quadriceps femoris (QF) and hamstrings. A reference marker was attached at the middle point between the great trochanter and lateral condyle of femur (Fig 1), and CSAs at 50% of thigh length were measured by manually tracing the outline of muscle tissue using a software (ImageJ, MIPAV; National Institutes of Health, Bethesda, USA). Visible adipose and connective tissues within individual muscles were excluded from the analysis. CSA values of four muscles were summed to provide QF and hamstrings CSAs, respectively (QF CSA: rectus femoris, vastus lateralis, vastus medialis and vastus intermedius; hamstrings CSA: biceps femoris short head, biceps femoris long head, semitendinosus and semimembranosus, Fig 1). The analysis of CSA for individual muscles was performed twice and the coefficient of variation (CV) of the two measured CSA values was calculated for each muscle. The analysis of CSAs for all individual muscles was performed by a single experienced examiner (AS) via the software (ImageJ, MIPAV; National Institutes of Health, Bethesda, USA), and the other two (TY and YK) agreed with his analyses.

#### Measurement of isometric knee extension and flexion torque

Participants performed unilateral (right side) maximal voluntary contraction (MVC) of isometric knee extension and flexion on a specially designed dynamometer (VTK-002, Vine, Japan). They sat on the device with the hip and knee joints fixed at 80° and 70° (anatomical position = 0°), respectively. Prior to the measurement, the participants completed a warm-up protocol consisting of submaximal contractions at 30, 50, and 80% of maximal effort to familiarise themselves with the measurement. Following a 1-min rest period, they performed two MVC attempts. It took approximately 3 seconds for the participants to attain a maximal value, and a minimum of 1-min recovery interval was set between MVC attempts to minimise any residual fatigue from the preceding attempt [25]. If the generated maximal torque differed by more than 10% between the attempts, they performed an additional one separated by a minimum of 1-min recovery until the two best values fell within the range of 10%. The torque signals were amplified by a strain amplifier (DPM-711B, Kyowa, Japan), converted via an AD converter (Power Lab, ADInstruments, Australia) and transferred to a computer at 1000 Hz with a low-pass filter (cut-off frequency, 10 Hz). The highest knee extension and flexion torque (i.e. the peak value observed at a single data point) derived from two or more attempts were adopted as MVC [26].

#### Measurement of bilateral leg extension power

Power of the lower limbs was assessed using a multi-joint leg extension apparatus (Anaeropress 3500, Combi, Japan). The load of leg extension was set to equal individuals’ body weight [27]. Participants sat back on the device, positioned their feet on the sliding foot plate with knee joint of 90°, and hip was securely strapped to the seat. After having performed several warm-up trials consisting of 30, 50 and 80% of maximal effort, maximal leg extension was performed five times interspersed with 15-s recovery between the attempts. They were asked to extend their legs as hard and as fast as possible, and the average of the two highest values was adopted as bilateral leg extension power as previously described [27–29]. The power was calculated as the product of a participant’s body weight (kgw) and the moving velocity (m/s) of the foot plate [27, 28].

#### Measurement of physiological parameters during a continuous ramp-incremental cycling

**Fig 2:**
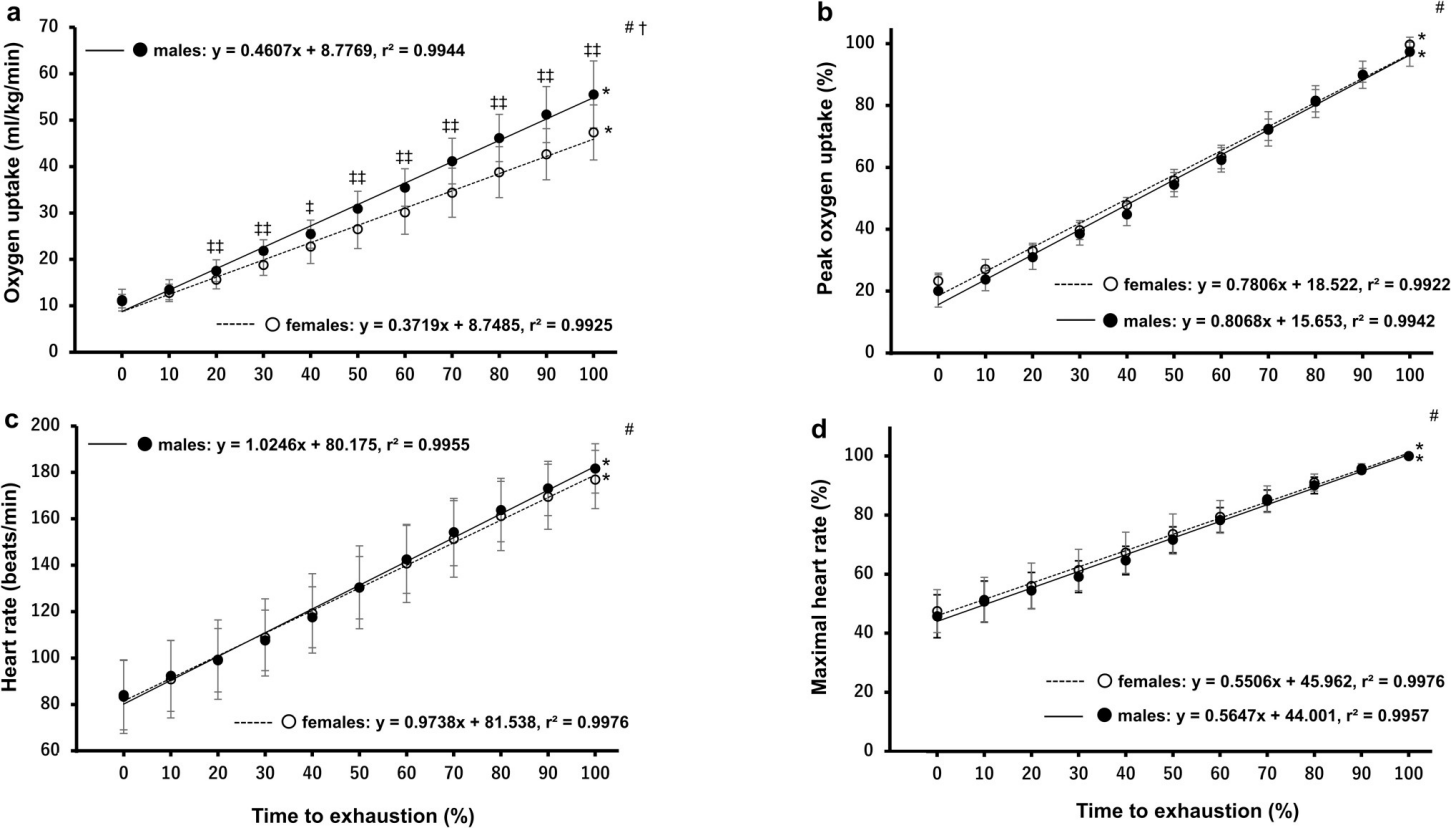
Time course changes of oxygen uptake and heart rate in response to the continuous ramp-incremental cycling test. Fig 2A and 2B show oxygen uptake response as a function of percentage of TTE expressed as absolute (left upper panel) and relative (right upper panel) responses, respectively. Fig 2C and 2D show heart rate response as a function of percentage of TTE expressed as absolute (left lower panel) and relative (right lower panel) responses, respectively. ^#^Indicates a main effect of time in the two-way mixed ANOVA (p < 0.001). ^†^Indicates a time-by-group interaction effect in the two-way mixed ANOVA (p < 0.001). *Indicates a main effect of time in the one-way repeated ANOVA (p < 0.001). ^‡‡^Indicates greater than female athletes (p = 0.0097 to 0.0002). ^‡^Indicates greater than female athletes (p = 0.017). Significant differences between each of timepoints are not shown for clarity.

Participants performed a continuous ramp-incremental (RI) exercise test to exhaustion on a cycle ergometer (Corival cpet, Lode B.V., Groningen, Netherlands) to determine peak oxygen uptake ($\dot{V}O_{2peak}$) via breath by breath analysis (Metalyzer®3B gas analyser, Cortex, Leipzig, Germany). Following a warm-up of 3-min cycling at 20W, the test commenced at an initial power output of 20W, with an additional 15W, 25W or 35W increase every minute until the participants could not maintain a speed of 70 rpm or until volitional exhaustion occurred despite strong verbal encouragement [30]. The increase in power output was determined according to individuals’ body size as well as the results obtained from knee strength and leg extension power measurements so that all participants reached exhaustion at a similar timepoint [31]. The ramp slope (i.e. W/s) of each athlete was then determined based on the linear relationship between the elapsed time and the work rate increment [32]. Exercise duration at exhaustion was recorded to the nearest second and defined as time to exhaustion (TTE). Respiratory gas exchange measures were averaged every 15 seconds with $\dot{V}O_{2peak}$, peak ventilation ($\dot{V}E_{peak}$) and peak respiratory exchange ratio (RER_peak_) being defined as the highest values over a 15-s period, while power output corresponding to $\dot{V}O_{2peak}$ was defined as maximal power putout (MPO) [32]. Heart rate was recorded throughout using a heart rate monitor (Polar Electro, Kempele, Finland) and was averaged every 15 seconds. Maximal heart rate (HR max) was defined as the highest heart rate recorded over a 15-s period. Oxygen pulse at $\dot{V}O_{2peak}$ was also calculated using the following equation: $\dot{V}O_{2}$ (ml/min)/HR (beats/min) [33, 34]. Moreover, as it has been shown that a better estimation of stroke volume is achieved when oxygen pulse is corrected for body mass [33, 34], O_2_ pulse was divided by mass in kilograms (ml/beat/kg) and multiplied by 100 as previously suggested [34]. In addition, in an attempt to determine the time course of changes in cardiorespiratory variables, $\dot{V}O_{2}$ and HR were plotted as a function of normalised time (i.e. percentage of TTE) using a cubic spline method in both absolute and relative (i.e. % $\dot{V}O_{2peak}$ and HR max) values throughout the RI test (Fig 2A–2D).

### Statistical analyses

All data are presented as means ± SD unless otherwise stated. The differences in physical and physiological characteristics between sexes were compared via two-tailed independent samples t-tests. Pearson’s product moment correlations were examined to assess interrelationship between the variables. In addition, to determine predictive variables for MPO, six independent variables ($\dot{V}O_{2peak}$, CSAs of QF and hamstring muscles, knee extension and flexion torque and bilateral leg extension power) were entered into a forward stepwise multiple regression model with MPO as the dependent variable, for males and females separately. The criteria used for entering and removing the stepwise regression model were the probability of F values of ≤ 0.05 and ≥ 0.10, respectively. To exclude the influence of body mass on the bivariate correlation and the stepwise regression model, all variables were normalised to body mass or two-thirds power of body mass in the case of CSA [35]. Variance inflation factor (VIF) was determined to check whether a predictor has a strong linear relationship with other predictor(s), while Durbin-Watson test was conducted to confirm the assumption of independence. Moreover, a two-way analysis of variance (ANOVA) with between (male vs. female) and repeated (time) factors was utilised to determine the time course of changes in $\dot{V}O_{2}$ and HR during the continuous ramp-incremental cycling. If a significant main effect of time was observed, a one-way repeated ANOVA was employed to determine the changes of $\dot{V}O_{2}$ or HR with time in each group. Furthermore, where the analysis revealed a significant time-by-group interaction effect, differences in $\dot{V}O_{2}$ or HR between the groups at each timepoint were compared by two-tailed independent samples t-tests. While a post-hoc Bonferroni multiple correction was also performed following the one-way repeated ANOVA, differences between each of timepoints are not shown for clarity as it is obvious that cardiorespiratory variables are increased with the increase in work rate (Fig 2). In all ANOVA tests, the Greenhouse-Geisser correction was used where the violation of sphericity was detected. Finally, 95% confidence interval (CI) and effect size (ES) were also reported where appropriate. ES was calculated as Cohen’s *d* by subtracting the mean value of the female athletes from that of the male athletes and dividing by the pooled standard deviation [36]. All statistics were run on IBM® SPSS® version 24.0 for Windows, and the level of significance was set at p < 0.05.

## Results

### Body composition

Male athletes were significantly taller and heavier than female athletes (height: 174.5 ± 7.6 vs. 163.4 ± 10.1 cm, p < 0.001; body mass: 69.5 ± 9.1 vs. 56.4 vs. 8.5 kg, p < 0.001), while they had significantly lower body fat percentage compared with female athletes (15.1 ± 2.9 vs. 22.3 ± 2.7%, p < 0.001).

### Anatomical cross-sectional areas of thigh muscles

CVs of QF and hamstrings CSAs between twice manually tracing measures for individual muscles were 2.1 ± 0.8% and 2.6 ± 1.3%, respectively. Male athletes had significantly greater QF CSA compared with female athletes in both absolute and relative values (absolute: 71.8 ± 9.8 vs. 52.6 ± 13.5 cm^2^, p < 0.001; relative: 4.26 ± 0.52 vs. 3.56 ± 0.72 cm^2^/kg^2/3^, p = 0.001). Likewise, the CSA of hamstrings was significantly greater in male than female athletes in both absolute and relative values (absolute: 28.6 ± 4.6 vs. 22.3 ± 4.3 cm^2^, p < 0.001; relative: 1.70 ± 0.26 vs. 1.52 ± 0.25 cm^2^/kg^2/3^, p = 0.036).

### Unilateral knee extension and flexion torque and bilateral leg extension power

**Fig 3:**
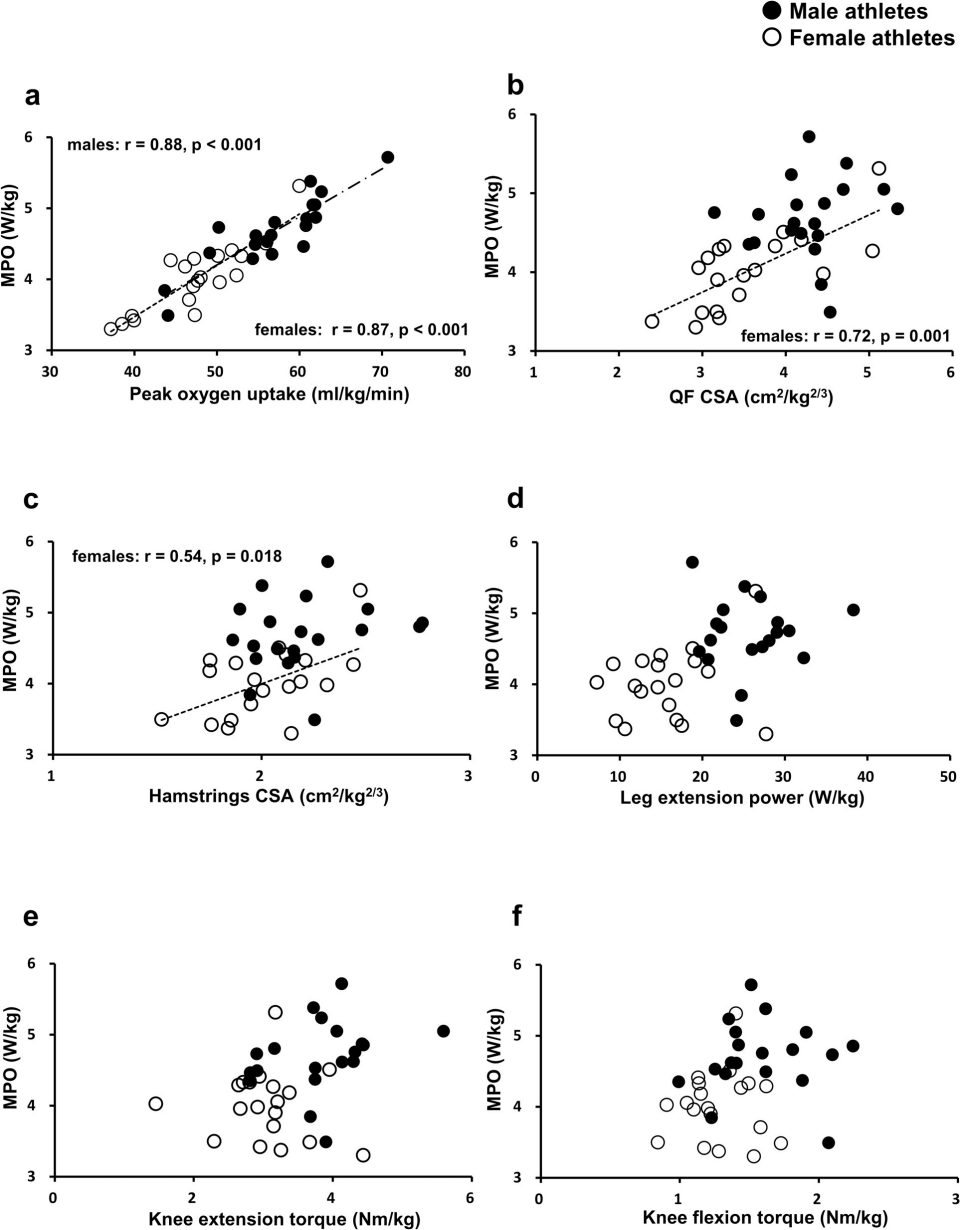
Correlations between maximal power output and (a) peak oxygen uptake, (b) QF CSA, (c) hamstrings CSA, (d) leg extension power, (e) knee extension torque and (f) knee flexion torque. MPO = maximal power output, QF = quadriceps femoris, CSA = cross-sectional area.

Male athletes had significantly greater unilateral knee extension and flexion torque and bilateral leg extension power compared with female athletes (Table 2). One male athlete refrained from performing the MVC and leg extension power tests due to a light hip injury. However, since he did complete all the other measurements, his data were included in the overall data (Tables 1 and 2) and the bivariate correlations between MPO and $\dot{V}O_{2peak}$, QF CSA and hamstrings CSA (Fig 3A–3C). On the other hand, his data were excluded from the bivariate correlations between MPO and leg extension power, KE torque and KF torque (Fig 3D–3F) in addition to the stepwise multiple regression analysis (Table 3).

**Table 2:** Physiological and performance characteristics of athletes.

Variables	Male athletes (n = 20)	Female athletes (n = 19)	ES
$\dot{V}O_{2peak}$ (ml/kg/min)	57.0 ± 6.6** (53.9–60.0)	47.5 ± 5.9 (44.7–50.4)	1.51
$\dot{V}E_{peak}$ (l/min)	143 ± 29** (129–156)	101 ± 26 (88–113)	1.53
RER_peak_	1.12 ± 0.03 (1.11–1.14)	1.10 ± 0.05 (1.08–1.12)	0.47
Maximal heart rate (beats/min)	183 ± 10 (178–188)	178 ± 13 (171–184)	0.44
Peak O_2_ pulse (ml/beat/kg)	31.8 ± 3.6** (30.1–33.5)	26.7 ± 3.3 (25.0–28.4)	1.48
MPO (W/kg)	4.7 ± 0.5** (4.4–4.9)	4.0 ± 0.5 (3.8–4.3)	1.32
Time to exhaustion (seconds)	727 ± 108 (676–777)	721 ± 167 (641–802)	0.04
Leg extension power (W/kg)	25.7 ± 4.9** (23.3–28.1)	15.7 ± 5.4 (13.1–18.3)	1.94
Knee extension torque (Nm/kg)	3.8 ± 0.7** (3.5–4.2)	3.0 ± 0.6 (2.7–3.3)	1.17
Knee flexion torque (Nm/kg)	1.6 ± 0.3* (1.4–1.7)	1.3 ± 0.2 (1.2–1.4)	1.03

HR data from one male and two female athletes were excluded due to a mechanical error. One male athlete did not perform knee strength and leg extension power tests due to a light hip injury.

** Significantly different from female athletes (p < 0.001).

* Significantly different from female athletes (p = 0.003). Numbers in the parentheses indicate 95% confidence interval of each variable. ES = effect size, $\dot{V}O_{2peak}$ = peak oxygen uptake, $\dot{V}E_{peak}$ = peak ventilation, RER_peak_ = peak respiratory exchange ratio, MPO = maximal power output

**Table 3:** Stepwise multiple regression analysis.

Group	Predictors	Regression coefficient	SE	95% CI	p	r^2^	Adjusted r^2^	VIF	Durbin-Watson
Males	$\dot{V}O_{2peak}$	0.067	0.009	0.049–0.085	< 0.001	0.78	0.77	1.000	2.116
Females									
Step 1	$\dot{V}O_{2peak}$	0.072	0.010	0.051–0.093	< 0.001	0.76	0.74	1.000	-
Step 2	$\dot{V}O_{2peak}$	0.057	0.010	0.035–0.078	< 0.001	0.76	0.74	1.436	2.245
	QF CSA	0.235	0.082	0.061–0.409	= 0.011	0.84	0.82	1.436	

Dependent variable: Maximal power output. Six independent variables ($\dot{V}O_{2peak}$, CSAs of QF and hamstring muscles, knee extension and flexion torque and bilateral leg extension power) were entered into a forward stepwise multiple regression model. Data from one male athlete were excluded from the analysis since he did not perform knee strength and leg extension power tests due to a light hip injury. $\dot{V}O_{2peak}$ = peak oxygen uptake, QF = quadriceps femoris, CSA = cross-sectional area, SE = standard error, CI = confidence interval, VIF = variance inflation factor

### Physiological parameters during the continuous ramp-incremental cycling

The ramp slopes for male and female athletes were 0.43 ± 0.07 W/s and 0.30 ± 0.08 W/s, respectively (males > females, p < 0.001). Male athletes had significantly greater $\dot{V}O_{2peak}$, O_2_ pulse, $\dot{V}E_{peak}$ and MPO than female athletes, while there were no significant differences in RER_peak_, HR max or time to exhaustion between the sexes (Table 2). When the cardiorespiratory variables were normalised to time to exhaustion, male athletes showed greater $\dot{V}O_{2}$ than female counterparts from 20% of TTE onwards (Fig 2A). In contrast, there was no difference in the time course changes of $\dot{V}O_{2}$ between the groups when it was expressed as % $\dot{V}O_{2peak}$ (Fig 2B). HR was similarly increased by male and female athletes throughout the test whether it was expressed as absolute or relative value (Fig 2C and 2D).HR data from one male and two female athletes were excluded due to a mechanical error.

### Correlation coefficients and stepwise multiple regression analysis

In male athletes, there was a significant correlation between MPO and $\dot{V}O_{2peak}$ (r = 0.88, p < 0.001, Fig 3A) and $\dot{V}O_{2peak}$ was selected as the single predictor of MPO by the stepwise multiple regression model (Table 3). In female athletes, there were significant correlations between MPO and $\dot{V}O_{2peak}$ (r = 0.87, p < 0.001, Fig 3A), the CSA of QF (r = 0.72, p = 0.001, Fig 3B) and the CSA of hamstrings (r = 0.54, p = 0.018, Fig 3C). The multiple regression model revealed that MPO was attributed to, in the order of importance, $\dot{V}O_{2peak}$ and the CSA of QF (Table 3). In both male and female athletes, significant correlations were not found between MPO and the parameters of lower extremity strength or power (Fig 3D–3F). Regression equations for male and female athletes obtained from the stepwise multiple regression analysis were as follows:

Male athletes: MPO (W/kg) = (0.067 x $\dot{V}O_{2peak}$ [ml/kg/min]) + 0.876

Female athletes: MPO (W/kg) = (0.057 x $\dot{V}O_{2peak}$ [ml/kg/min]) + (0.235 x QF CSA [cm^2^/kg^2/3^]) + 0.493

## Discussion

The novel findings of the present study are that while $\dot{V}O_{2peak}$ is the main determinant of maximal incremental power output regardless of sex or fitness level, the size of QF also plays a role in whole-body maximal aerobic performance in female athletes. It has been established that the ability of oxygen transport is the primary limiting factor for exercise tolerance during whole-body maximal aerobic exercise [1, 5, 13, 14]. In line with previous findings [5], this study also showed that $\dot{V}O_{2peak}$ (a parameter of oxygen transport) primarily determined MPO. While stroke volume has been shown to increase until exhaustion in endurance trained athletes, the increase of SV may reach a plateau at 40 to 50% of $\dot{V}O_{2peak}$ in untrained individuals [8, 9, 22], suggesting that SV response to exercise is fitness-level dependent. Therefore, especially in less endurance-trained individuals, it could be assumed that the importance of non-aerobic factors (e.g. lower-limb muscle size, strength and power) may increase where the rate of oxygen supply does not meet its demand at working muscles. Although we did not directly measure SV in the current study, lower $\dot{V}O_{2peak}$ and oxygen pulse in female athletes would suggest their limited capacity of oxygen transport, thereby increasing the degree of dependence on thigh muscles with the work rate increment. Akima et al. [37] observed high correlations (r = 0.71 to 0.83) between power output of repeated cycle sprints and a magnitude of muscle activation of QF assessed by changes in T2 values from pre- to post-sprint using T2-weighted MRI. Moreover, they also confirmed lower but significant correlations between the power output and changes in T2 values of hamstrings (r = 0.40 to 0.58) [37]. Their findings are somewhat in line with the current study where the CSAs of QF and hamstrings were significantly correlated with MPO in female athletes (r = 0.72 and 0.54 for QF and hamstrings, respectively. Fig 3B & 3C). While they employed repeated supramaximal sprints as opposed to incremental cycling, their findings would suggest an important role of thigh muscles (especially QF) in power generation during high-intensity cycling. Although knee extension strength or leg extension power was not selected as a predictive variable of MPO in either group, we might have seen a different result if strength or power was assessed in a modality-specific testing condition (i.e. cycling) [16, 38, 39] or the parameters were determined at given joint angles and velocity closely associated with upright cycling [17].

While the ramp slope was significantly greater in male than female athletes during the continuous ramp-incremental test (0.43 ± 0.07 W/s vs. 0.30 ± 0.08 W/s, p < 0.001), there was no significant difference in time to exhaustion or HR max between the groups (Table 2). Similar slopes of increment (0.28 and 0.42 W/s) were previously employed by Adami et al. [32] during incremental stepwise ramp test (IRST) protocols, and $\dot{V}O_{2\max}$ and HR max elicited during the IRST protocols were almost identical compared with those of the most classical protocol (i.e. incremental intermittent Åstrand-type test). This indicates that the continuous RI protocols employed in the current study similarly and properly induced peak cardiorespiratory capacities for both male and female athletes.

In addition to the ability of circulatory system to deliver oxygen to working muscles, exercise tolerance can also be limited by perception of effort especially when tested individuals are not informed of exercise duration (i.e. when to stop) [38, 40, 41]. Interestingly, it has been demonstrated that, when young healthy adults were required to perform a short all-out effort sprint immediately after an incremental exercise to exhaustion or time to exhaustion test, they were able to generate power, at levels far above that produced at exhaustion (task failure) in the preceding exhaustive exercise [38, 39]. These findings suggest that a large functional reserve remains in the muscles to generate power even at task failure, and peripheral fatigue does not necessarily cause exhaustion during high-intensity aerobic exercise [38, 39]. Instead, it has been suggested that fatigue occurs to avoid a catastrophic failure of homeostasis in the exercising muscles [42], and maximal power that the muscles can produce potentially changes the time to exhaustion with increased and decreased power production capacities causing later and earlier onset of exhaustion, respectively [38]. Considering the differences between male and female athletes in the parameters associated with lower extremity power production (e.g. QF CSA, isometric knee extension torque and maximal leg extension power, even after normalised to body mass), functional reserve in the exercising muscles to generate power at exhaustion also seems to have been different between the sexes in the current study [38, 39]. Lower capacity in muscle power production (and less functional reserve at exhaustion) may have exacerbated perception of effort in female athletes [38]. If this is the case, it could be argued that, in the current study, the size of QF (one of the parameters associated with muscle power production) potentially limited exercise tolerance by influencing perception of effort in female athletes. In contrast to the current study, it has been shown that females possess greater fatigue resistance in knee extensors than males during single-limb exercises [43–45], which has been attributed to lesser deoxygenation of the knee extensors in females during fatiguing tasks [44]. Furthermore, there was no difference in time to task failure during whole-body (cycling) trials between male and female participants when the exercise intensity was normalised to the power-duration relationship (i.e. critical power [CP]) [46]. Moreover, the females showed lesser deoxygenation of vastus lateralis during the fatiguing cycling trials at 90% and 110% of CP and experienced lesser reductions in knee-extensor contractile function following both trials [46]. The discrepancies between the current study and the previous studies are likely explained by the method utilised (i.e. absolute vs. relative intensity). Indeed, the females produced approximately 30% lower CP compared with males (179 ± 32 vs. 260 ± 28 W, p < 0.001) in the study by Ansdell et al. [46]. Therefore, it has yet to be determined whether a similar phenomenon would be observed when comparison is made at absolute or maximal intensity.

Another candidate that possibly explains the findings of the current study is sex-related differences in the degree of leg muscle pain [47]. In the study by Cook et al. [47], the pain ratings were higher and increased at a faster rate in young moderately active females than males as the exercise intensity increased during incremental cycling to exhaustion [47]. The authors attributed the sex difference to the amount of muscle mass by stating that their female participants were required to recruit a greater percentage of the total available muscle fibres compared with male counterparts at any given absolute intensity [47]. In addition, since female participants possessed lower $\dot{V}O_{2peak}$ in their study, they also argued that the females were likely to have relied more on anaerobic metabolism to generate energy at lower exercise intensities, which could also contribute to the greater pain sensation in their female participants [47]. Their arguments are likely supported by the current study where female athletes consumed less oxygen relative to their body mass than males from 20% of TTE onwards (Fig 2A). Although we did not investigate the pain ratings, lower $\dot{V}O_{2peak}$ together with smaller thigh muscle size may have caused a greater pain sensation in the females as the exercise intensity increased. This could be another reason why the OF size was one of the limiting factors for MPO in the female athletes.

### Limitations

There are several limitations in the current study. Firstly, although QF size was chosen as a predictive variable of MPO in female athletes only, it is not clear whether this phenomenon was originated from sex difference itself since there were significant differences in cardiorespiratory fitness level and lower extremity power production capacity between male and female athletes. Secondly, considering that previous studies showed a sport-specific physiological response to incremental exercise [48], we cannot rule out the possibility that the heterogeneity of sports affected the results of this study. Nevertheless, it should be noted that both groups included only one athlete who was accustomed to upright cycling (i.e. one male triathlete and one female cyclist, respectively, Table 1), indicating that there was no difference between the groups regarding the degree of proficiency in upright cycling. Another limitation is the lack of blood sample collection. Peak blood lactate concentration following exhaustive exercise is thought to reflect anaerobic glycolytic capacity [49], and it differs among different athletic populations [50]. Thus, this parameter could have explained the differences between the sexes or among sporting disciplines in the current study. Moreover, since by-product of anaerobic metabolism could contribute to the pain sensation during and following exhaustive exercise [47], the collection and analysis of biochemical markers such as hydrogen ions and bradykinin may have accounted for some of the differences observed between the sexes in this study [47]. What is more, while there was no significant difference in age between male and female athletes, years of experience in the selected sporting disciplines were significantly longer in male than female athletes (12 ± 3 vs. 9 ± 3 years, p = 0.005). Longer years of competitive (and associated training) experience in the male athletes may indicate their higher pain or fatigue tolerance against the exhaustive exercise when compared with the female counterparts [47]. However, it was not possible to confirm this assumption in the current study as we did not obtain the pain ratings or the rate of perceived exertion. Finally, we did not collect the data regarding our athletes’ lower-limb dominance when measuring knee extension and flexion torque. This was because the dynamometer (VTK-002, Vine, Japan) was pre-set for the measurement of right leg, and we could not change the settings according to their lower-limb dominance. This could be another limitation considering that lower-limb strength can be significantly different between dominant and nondominant legs [51]. Nevertheless, we recall that the majority of our participants were right-footed through oral communication prior to the measurement. Moreover, our participants also performed the bilateral leg extension power test, which should somewhat compensate the lack of a bilateral strength measurement. Taken together, future research should elucidate the influence of non-aerobic factors on maximal aerobic exercise performance with the control of sporting discipline, training background, aerobic fitness level and the capacity of lower extremity power production.

## Conclusions

In conclusion, this study has shown that while oxygen transport capacity is the main determinant of maximal incremental power output in both sexes, thigh muscle size also has a role in the limit of tolerance during whole-body maximal aerobic exercise in the female athletes. Possible reasons for the observed phenomenon are that compared with the male athletes, the female athletes possessed less ability of circulatory system to meet an increasing oxygen demand at working muscles, and less functional reserve in the muscles to generate power at exhaustion, both of which potentially increased the role of thigh muscles in the whole-body maximal aerobic exercise in this group. Nevertheless, the generalisability of the current study may be limited considering the heterogeneity of sporting disciplines.

## Supporting information

S1 File Raw data of all parameters_R3. (XLSX)

S2 File Raw data of cardiorespiratory responses as a function of normalised time during the ramp-incremental cycling test. (XLSX)
